# P-2. Public Health and Economic Impact of Implementing Gender-Neutral Vaccination with the 4-Valent Human Papillomavirus Vaccine in Costa Rica

**DOI:** 10.1093/ofid/ofae631.213

**Published:** 2025-01-29

**Authors:** Juan Carlos Orengo, Ana Marisol Rendon, Bruna Cristina Lima, Andrew Pavelyev, Vincent Daniels, Kunal Saxena, Wilberth Herrera-Solano, Cintia I Parellada

**Affiliations:** MSD (IA) LLC, Guaynabo, Puerto Rico; MSD Panama, Panama, Panama, Panama; MSD Brazil, São, Sao Paulo, Brazil; Merck&Co.,Inc., Rahway, New Jersey; Merck&Co.,Inc., Rahway, New Jersey; Merck&Co.,Inc., Rahway, New Jersey; Caja Costarricense de Seguro Social, San Jose, San Jose, Costa Rica; MSD Brazil, São, Sao Paulo, Brazil

## Abstract

**Background:**

In 2019, the quadrivalent human papillomavirus female only-vaccination (4vHPV-FOV) was introduced for 10-year-old girls in the Costa Rican National Immunization Program. This analysis aims to estimate the public health impact and the incremental cost-effectiveness of switching from a 4vHPV-FOV to a 4vHPV-GNV in Costa Rica.

**Table 1.**
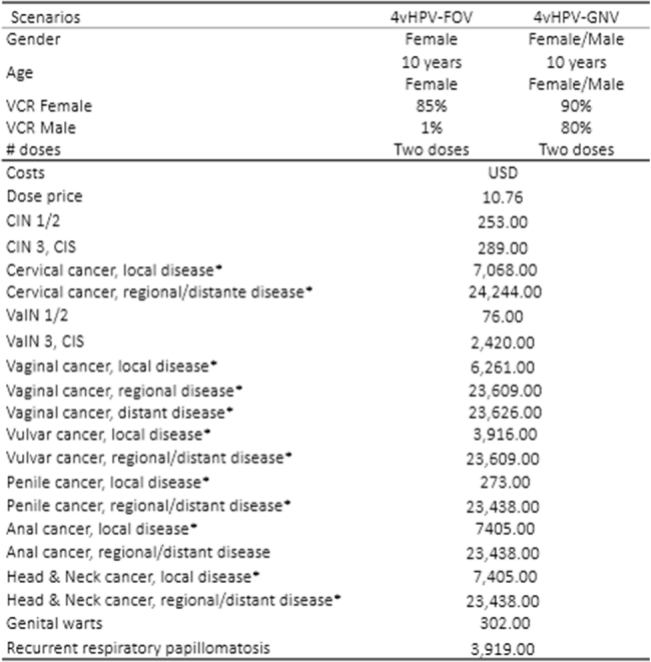
Main parameters used in the model

**Methods:**

A validated dynamic model of HPV disease transmission was calibrated to simulate the natural history of HPV infection and attributable disease burden in Costa Rica. The model assumed a two-dose schedule over a 100-year time horizon, lifetime immunity after vaccination, continuous cytology screening, and herd immunity. The outcomes measured were incremental averted cases and deaths of cervical, vaginal, vulvar, anal, penile, head & neck HPV-attributable cancers; recurrent respiratory papillomatosis, genital warts, and cervical intraepithelial neoplasia (CIN); and incremental cost-effectiveness ratio (ICER) expressed in quality-adjusted life years. Outcomes and costs were discounted at 3% annually rate. Costa Rica specific data were used for calibration (Table 1). The WHO definition of cost-effectiveness was used to define the threshold of highly cost-effective (ICER < 1 GDP/per capita or US$13,365.4 for Costa Rica).

**Table 2.**
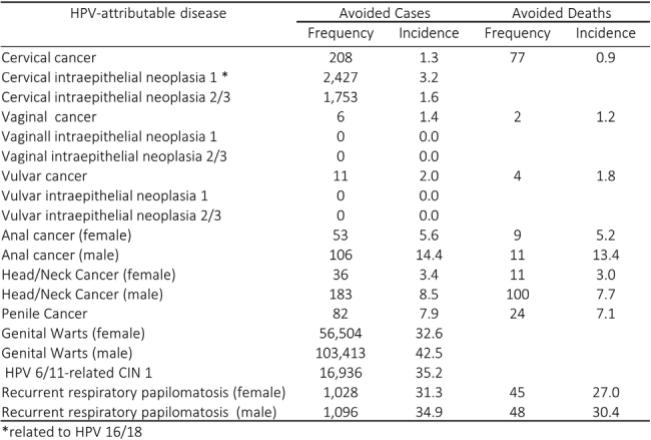
Additional avoided cases/cumulative percent reduction and deaths/mortality percent reduction of HPV-attributable diseases and cancers with 4vHPV-GNV strategy relative to 4-HPV-FOV over 100 years in Costa Rica.

**Results:**

After 100 years, the 4vHPV-GNV strategy was projected to avert an additional 206,211 cases and 481 deaths compared to 4vHPV-FOV. The greatest reductions in burden and costs were due to HPV-6/11-attributable diseases (Table 2 and Fig. 1). For HPV 16 and 18-attributable cancers, the greatest reductions in burden were observed in cervical cancer, CIN 1 and 2/3 for females, and head & neck and penile cancers for males (Fig 1 and 2). 4vHPV-GNV was highly cost-effective with an ICER of US$ 2,166.

**Figure 1.**
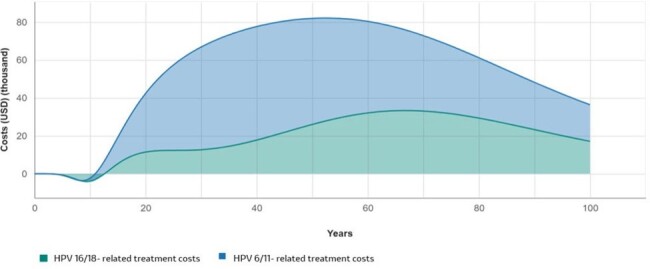
Estimated healthcare costs avoided over 100 years by HPV genotype in Costa Rica

**Conclusion:**

In Costa Rica, switching from a 4vHPV-FOV to a 4vHPV-GNV strategy is projected to be a cost-effective intervention, providing a substantial public health impact in both genders. The 4vHPV-GNV scenario would result in faster and greater reductions in the incidence of HPV 6/11/16/18-attributable diseases and cancers compared to the 4vHPV-FOV.

**Figure 2.**
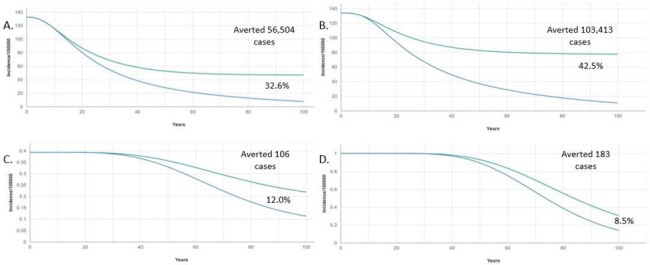
Estimated HPV 6/11/16/18-attributable disease and cancer incidence over 100 years in Costa Rica A.Genital warts among females, B. Genital warts among males, C. Anal cancer among males, D. Head & Neck cancers among males

**Disclosures:**

**Juan Carlos Orengo, MD, MPH, PhD**, Merck & Co., Inc: Employee|Merck & Co., Inc: Stocks/Bonds (Private Company) **Ana Marisol Rendon, MD, MS**, Merck & Co., Inc: Employee|Merck & Co., Inc: Stocks/Bonds (Private Company) **Bruna Cristina Lima, n/a**, Merck & Co., Inc: Employee **Andrew Pavelyev, n/a**, HCL America, Inc.: emplo **Vincent Daniels, PhD**, Merck & Co., Inc: Employee|Merck & Co., Inc: Stocks/Bonds (Private Company) **Kunal Saxena, PhD**, Merck & Co., Inc: Stocks/Bonds (Private Company) **Wilberth Herrera-Solano, MD**, Consultant: Consultant **Cintia I. Parellada, MD, PhD**, Merck & Co., Inc: Employee|Merck & Co., Inc: Stocks/Bonds (Private Company)

